# Health and Well-Being in Surviving Congenital Heart Disease Patients: An Umbrella Review With Synthesis of Best Evidence

**DOI:** 10.3389/fcvm.2022.870474

**Published:** 2022-06-10

**Authors:** Lucia Cocomello, Kurt Taylor, Massimo Caputo, Rosie P. Cornish, Deborah A. Lawlor

**Affiliations:** ^1^MRC Integrative Epidemiology Unit, Population Health Sciences, Bristol Medical School, University of Bristol, Oakfield House, Bristol, United Kingdom; ^2^Bristol Heart Institute, Bristol, United Kingdom

**Keywords:** congenital heart disease, long term, umbrella review, adult, health and well-being

## Abstract

**Background:**

Advances in the management of congenital heart disease (CHD) patients have enabled improvement in long-term survival even for those with serious defects. Research priorities (for patients, families and clinicians) have shifted from a focus on how to improve survival to exploring long-term outcomes in patients with CHD. A comprehensive appraisal of available evidence could inform best practice to maximize health and well-being, and identify research gaps to direct further research toward patient and clinical need. We aimed to critically appraise all available published systematic reviews of health and well-being outcomes in adult patients with CHD.

**Methods:**

We conducted an umbrella review, including any systematic reviews that assessed the association of having vs. not having CHD with any long-term health (physical or mental), social (e.g., education, occupation) or well-being [e.g., quality of life (QoL)] outcome in adulthood (≥18-years).

**Results:**

Out of 1330 articles screened, we identified five systematic reviews of associations of CHD with adult outcomes. All but one (which studied QoL) explored health outcomes: one cardiovascular, two mental, and one mortality after transplant. CHD patients had a higher risk of stroke, coronary heart disease and heart failure, with the pooled relative risk (RR) for any outcome of 3.12 (95% CI: 3.01 to 3.24), with substantial heterogeneity (I^2^ = 99%) explained by the outcome being studied (stronger association for heart failure) and geography (stronger in Europe compared with other regions). CHD patients had a higher risk of anxiety (OR = 2.58 (1.45 to 4.59)], and higher mean scores for depression/anxiety symptoms (difference in means = −0.11 SD (–0.28 to 0.06), I^2^ = 94%)]. Compared with patients having a cardiac transplant for other (non-CHD) diseases, CHD patients had higher short-term mortality (RR at 30-days post-transplant = 2.18 [1.62 to 2.93)], with moderate heterogeneity (I^2^ = 41%) explained by previous surgery (higher mortality with prior Fontan/Glenn operation). All domains of QoL were lower in patients with Fontan’s circulation than non-CHD adults.

**Conclusion:**

Adults with CHD have poorer cardiovascular, mental health and QoL outcomes, and higher short-term mortality after transplant. The paucity of systematic reviews, in particular for outcomes such as education, occupation and lifestyles, highlights the need for this to be made a priority by funders and researchers.

**Systematic Review Registration:**

[www.crd.york.ac.uk/prospero], identifier [CRD42020175034].

## Introduction

Congenital heart diseases (CHD) are among the most common types of congenital anomalies, affecting between 6 and 8 individuals per 1000 live births (1). Historically, CHDs have been considered solely a pediatric disorder, given that only a minority of patients with moderate and severe CHD reached adulthood. However, advances in the management of this high-risk subgroup have enabled substantial improvement in long-term survival even for those with serious cardiac defects, with more than 90% of patients with CHD reaching adult life (2). As more CHD patients live longer, research priorities need to shift from a primary focus on how to improve survival to include research on the health and well-being of those surviving through childhood and into adulthood. Patients, their families and charities who support them have identified the need for research that assesses the extent to which CHD patients can expect to live healthy, productive lives in comparison to those without CHD, including differences in health, reproductive capacity, behaviors such as physical activity and in educational attainment and career prospects (3, 4). Further ongoing initiatives (e.g., The James Lind Alliance, a United Kingdom non-profit initiative that includes patients with CHD, their carers and clinicians) are expected to identify research priorities for children and adults with CHD by bringing together patients, carers and clinicians (5). Consequently, research around long-term outcomes in patients with CHD has become increasingly important.

It is difficult to get an overall picture of what the key risks to future health and well-being are. A comprehensive appraisal of available evidence could provide information for patients, their families and clinicians on important aspects of their adult life and areas where targeted interventions, such as additional educational support, or earlier monitoring to prevent diseases, might be valuable. An up-to-date review of current evidence can also provide guidance on future research needs around the long-term consequences of CHDs.

Umbrella reviews are systematic reviews of existing systematic reviews that can produce holistic evidence and identify important gaps in the literature (6). As systematic reviews are only recently emerging for long-term outcomes in CHDs, an umbrella review could be important in identifying outcomes where there is sufficient robust evidence to reassure patients (e.g., of no or little risk) or provide guidance (such as additional educational support or clinical monitoring) and identify research gaps.

To our knowledge, an umbrella review of long-term outcomes beyond survival in adults with CHD has not been undertaken to date. We therefore aimed to critically appraise available systematic reviews of health and well-being of adult patients with CHD. We aimed to include a broad range of outcomes and therefore did not pre-specify specific outcomes. Our hope was that we would identify systematic reviews covering educational achievement, quality of life (QoL), psychological functioning, neurodevelopment, reproduction/pregnancy, social and behavioral outcomes (e.g., physical activity, occupation) as well as outcomes reflecting cardiovascular health. Ultimately, we aimed to use findings from an umbrella review to develop recommendations for future research and clinical practice.

## Methods

The present work was developed according to current recommendations for umbrella reviews (6). The protocol was registered in PROSPERO (registration Number CRD42020175034).

### Inclusion Criteria

#### Study Design

Systematic reviews with or without meta-analyses were included. Studies were considered a systematic review if they met the following criteria: (i) the research question was clearly stated; (ii) a reproducible search strategy was presented (e.g., naming of databases, platforms/engines, search date and complete search strategy); (iii) inclusion and exclusion criteria were stated; (iv) selection (screening) methods were well defined; (v) study quality/risk of bias was critically appraised; (vi) information about data analysis and synthesis were provided (7).

#### Population

The target population was adults, defined as anyone aged 18 years or above. If reviews defined themselves as exploring outcomes in adults but used a lower age threshold (e.g., 16 years) we included those studies and tried to seek results for those only 18 years or older. The exposure was having a CHD vs. not. This was defined as born with any type of CHD, whether diagnosed antenatally, at birth or later in life. We included reviews of studies with any type of non-CHD comparison group and, in summarizing findings, considered the different sources of bias in relation to different comparison groups (see Risk of bias below).

#### Outcomes

In accordance with recommendations for umbrella reviews and previous umbrella reviews with other research aims (8), we did not pre-specify specific outcomes of interest. This is because the aim of umbrella reviews in general and our specific aim here was broad-reaching, i.e., to identify all systematic reviews of the association of CHD with any long-term health (physical or mental), social (e.g., education, occupation) or well-being (e.g., QoL) outcome in adulthood, so that we could summarize current evidence on associations and identify research gaps.

### Search Strategy

A comprehensive search of electronic databases MEDLINE (*via* PubMed), EMBASE, SCOPUS, PsycINFO and Cochrane library was conducted to identify relevant systematic reviews published between the beginning of each database and April 2020 without language restrictions. We also manually screened reference lists of the retrieved systematic reviews to identify any additional relevant systematic reviews. Searches were re-run prior to the final submission of the paper (October 2021). Supplementary Appendix 1 provides a detailed search strategy for the Medline, EMBASE, PSYCINFO, SCOPUS and the Cochrane Database of Systematic Reviews. The search strategy was developed around the key terms: [(Systematic Review [All]) or (meta-analysis[All])] and [(tetralogy of Fallot [TIAB]) or (pulmonary stenosis [TIAB]) OR (pulmonary valvar stenosis[TIAB]) or (congenital heart disease[TIAB]) or (congenital heart [TIAB])or (congenital cardiac disease [TIAB]) or (congenital heart defect [TIAB]) or (congenital heart malformation [TIAB]) or (ACHD [TIAB]) OR (GUCH [TIAB)] or (fontan circulation [TIAB]) or (cavo-pulmonary connection [TIAB]) or (univentricular heart [TIAB]) or (hypoplastic left heart syndrome [TIAB]) or (single ventricle [TIAB]) or (Norwood Procedure [TIAB]) or (double inlet left ventricle [TIAB]) or (double outlet right ventricle [TIAB]) or (Truncus arteriosus [TIAB]) or (ebstein [TIAB]) or (tricuspid atresia [TIAB]) or (ventricular septal defect [TIAB]) or (atrial septal defect [TIAB]) or (transposition of great arteries [TIAB]) or (transposition of great vessels[TIAB]) or (arterial switch [TIAB]) or (Senning [TIAB]) or (Mustard [TIAB]) or (aortic coarctation [TIAB])or (Interrupted aortic arch [TIAB])or (atrioventricular septal defect[TIAB]) or (total anomalous pulmonary venous connection [TIAB]) or (partial anomalous pulmonary venous connection [TIAB]) or (TAPVC [TIAB]) or (Cor triatriatum [TIAB]) or (Ross [TIAB]) or (Anomalous coronary artery [TIAB]) or (patent ductus arteriosus [TIAB])].

Furthermore, a librarian performed a separate search for individual clinical studies (with at least 500 cases) on each specific topic published after the reviews we identified (Supplementary Appendix 2). However, due to the large number of publications, these were not analyzed, and the search outcome is reported as a supplement.

### Study Selection and Data Extraction

Two authors (LC and KT) independently screened the titles and abstracts to exclude publications that did not meet our inclusion criteria. After selecting systematic reviews for inclusion, they then independently extracted relevant data according to a prior agreed form (Supplementary Appendix 3). Data extracted included: author, year of publication, number of studies included, number of participants and cases included for each study and each analysis, outcomes assessed, findings, subgroup analyses, confounders controlled for and assessment of heterogeneity. The study used two data extraction tools (one for selecting studies to be excluded and one for extracting data) developed *a priori*. LC and KT were blinded to each other’s decisions. Disagreements between them were resolved by asking one or more of the other authors to extract data (using the same form) for specific papers and then discussing results from all the extractions. If systematic reviews or meta-analyses examined more than one health outcome of interest, data for each outcome was recorded separately in the extraction process. Review authors were contacted for additional data where necessary.

### Risk of Bias Assessment

One author (LC) performed the risk of bias assessment with all the results checked by another author (KT). We assessed the risk of bias of the included systematic reviews using the ROBIS tool. ROBIS is a tool developed for assessing the risk of bias in systematic reviews regarding interventions, diagnosis, prognosis and etiology (9). The tool includes three phases. Phase 1 assesses the relevance of the study. Phase 2 identifies concerns with the review process across four domains (study eligibility criteria, identification and selection of studies, data collection and study appraisal, synthesis and findings). Phase 3 judges the overall risk of bias and summarizes the concerns identified during the Phase 2 assessment.

### Data Synthesis

We presented data from the original papers. Where a meta-analysis was carried out, we reported the pooled effect estimate with 95% confidence interval. Results were summarized with a narrative synthesis, forest plot and summary tables describing review characteristics and findings. Results from subgroup analyses or meta-regression analyses - when presented - were commented on.

## Results

### Literature Search

**FIGURE 1:**
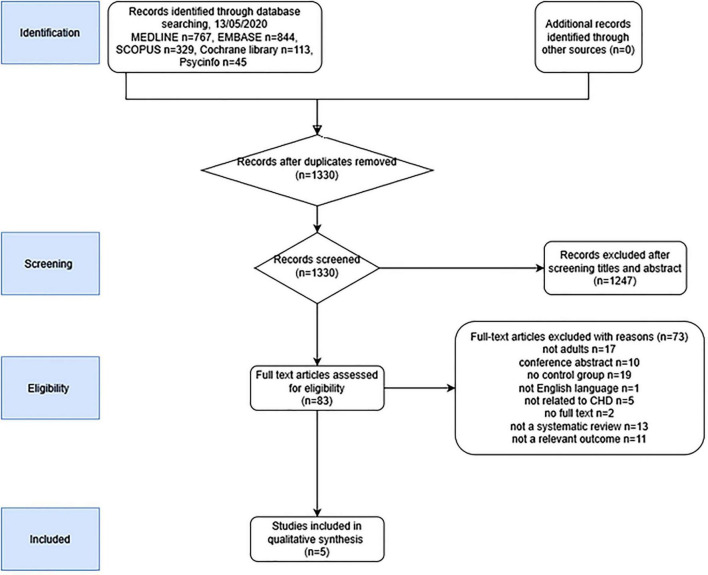
PRISMA flow chart of articles included.

Figure 1 summarizes the study selection process. In total, 1,330 unique citations were identified after an initial search. Of these, 1,247 were excluded after screening titles and abstracts. The full text of the remaining 83 articles were reviewed, and a further 78 excluded (key reasons for exclusions are provided in Supplementary Table 1) and the remaining five were included in the umbrella review.

Table 1 summarizes the characteristics of the five systematic reviews meeting our inclusion criteria. Three included studies of patients with any CHD compared to those with no CHD and explored associations with cardiovascular (one review) and mental health (two reviews) outcomes. One compared mortality after a cardiac transplant between CHD patients and those receiving a transplant for other diseases. One compared quality of life between CHD patients with a Fontan’s circulation (i.e., patients treated for single ventricle disease with a series of operations across childhood that allow oxygenated and deoxygenated blood to be separated in the heart) to non-CHD adults. We summarize the results for each outcome separately below.

**TABLE 1:** Overview of studies.

References	Exposure		Outcome	Inclusion criteria	Age at outcome assessment	Risk of bias/quality assessment (author’s conclusion)
	CHD definition	Comparison groupY=				
Wang et al. (10)	Any CHD *N* = 81,137	Adults without CHD selected from clinical records or health insurance databases *N* = 603,063	CVD defined as any of the following events: stroke, coronary artery, heart disease, heart failure, and cardiac arrest. Stroke was defined as any acute cerebrovascular event, composite stroke, stroke unspecified, and stroke/transient ischemic attack. Coronary artery heart disease was defined as ischemic heart disease, acute myocardial infarction, and coronary artery disease	Cohort study. Chinese or English language. Reported on CVD among patients with CHD.	Adults, adults and children, children	Study quality was generally good assessed using the Newcastle-Ottawa Scale. In particular overall sample representativeness, methods of ascertaining CHD and CV outcomes, and the description of the follow-up time.
Marshall et al. (14)	Fontan’s procedure* *N* = 197–346 numbers vary depending on specific outcomes	Healthy control sample or normative sample *N* = 327–2137 numbers vary depending on specific outcomes	Health related quality of life measured with the 36-item short-form health survey (SF-36)	All study designs and comparison group types Used a validated, quantitative self- or proxy- reported HRQOL measure English- language, peer- reviewed format	Mean patient age ranged from 20.7 to 27 years	Risk of bias assessments were performed using the 14-item criteria proposed by Kmet et al. (22), Risk of bias was low, and no studies were excluded because of bias.
Jackson et al. (11)	Any CHD *N* = 3,723	Healthy controls or normative sample N = not provided anywhere in the paper	Emotional functioning was defined as psychological symptoms, including symptoms of depression (i.e., feeling down, loss of energy, irritability, etc.) and anxiety (i.e., nervousness, worry, tension, etc.)	Studies that used a measure of emotional functioning, such as symptom-based assessment tools (e.g., Beck Depression Inventory) or quality of life surveys that had subscales measuring emotional functioning (e.g., The Medical Outcomes Study 36-Item Short-Form Health Survey – Mental Health Subscale) Participants > 14 years old Had a sample with < 10% with a genetic disorder	Patients age ranged from 13 to 87 years	The quality of each study was rated by using a modified instrument by Downs and Black (23), the author reported study quality did not moderate the effect sizes reported by the observed studies.
Secinti et al. (12)	Any CHD *N* = 999	Healthy control *N* = 229	adult emotional problems (i.e., depression, anxiety and unspecified emotional symptoms).	Longitudinal prospective, cross-sectional, or case-control designs Published in an English- language Participants > 16 years old	Mean age 27.7 years	The quality of each study was assessed using the Strengthening the Reporting of Observational Studies in Epidemiology (STROBE) guidelines, STROBE range from 63.6 to 66.7%
Doumouras et al. (24)	Any adult CHD recipient of a cardiac transplant *N* = 64–856, varies for different meta-analyses	Non-CHD adult recipients of a cardiac transplant *N* = 3,420–42,826, varies for different meta-analyses	Post cardiac transplant outcomes: mortality at 30 days, 1 year, 5 years and 10 years and cause-specific mortality	Observational Post-transplant outcome Participants ≥ 18 years old	Mean patient age ranged from 18 to 39 years	Risk of bias assessment, defined by the grade of recommendation, assessment, development and evaluation, highlighted risk of bias due to report in an unadjusted manner and unclear about length of follow up.

*Fontan’s procedure would be done in single ventricle disease.

*Y= As defined by authors.*

*CHD, congenital heart disease; HRQOL, health-related quality of life.*

### Cardiovascular Disease

**FIGURE 2:**
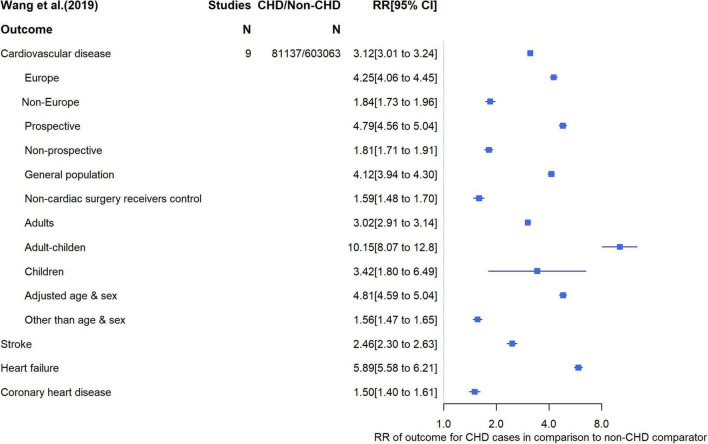
Relative risk for cardiovascular outcome and subgroup analysis.

We found one systematic review and meta-analysis exploring the association of being a CHD patient with risk of CVD, which pooled 9 cohort studies including a total of 684,200 participants (*N* = 81,137 CHD cases, *N* = 603,063 non-CHD) (10). CVD was defined as a composite of any study assessing stroke, coronary artery heart disease, heart failure, and cardiac arrest. Stroke was defined as any acute cerebrovascular event, composite stroke, stroke unspecified, and stroke/transient ischemic attack. Coronary artery heart disease was defined as ischemic heart disease, acute myocardial infarction, and coronary artery disease. In all studies, the association between CHD and risk of CVD was adjusted for age and sex; three studies adjusted for additional risk factors such as ethnicity, smoking and education. The relative risk (RR) in individual studies ranged from 1.48 to 10.76 across the range of different CVD outcomes. The pooled RR for any CVD outcome comparing CHD to non-CHD patients was 3.12 (95% CI: 3.01 to 3.24), with substantial between study heterogeneity (I^2^ = 99%). When outcomes were analyzed separately, the strongest association was found for heart failure (RR = 5.89 [5.58 to 6.21]; I^2^ = 93%; Figure 2). Geographic region (higher risk in European countries compared with other regions) and age (higher risk in studies including both adults and children compared to studies including adults or children only) were key sources of between study heterogeneity (Figure 2). The majority of the ROBIS tool criteria were at low risk of bias, with the exception that only studies written in English or Chinese were included.

### Mental Health

**FIGURE 3:**
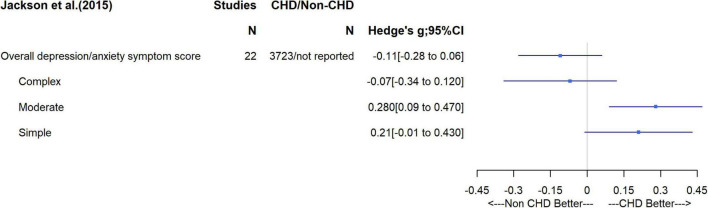
Differences in mean depression/anxiety symptom score and subgroup analysis on complexity of disease.

**FIGURE 4:**
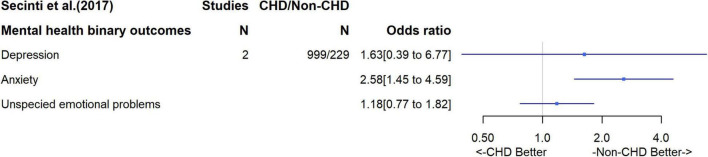
Odds ratio for mental health binary outcomes.

Two systematic reviews of mental health outcomes were identified. One explored the incidence of symptoms of depression or anxiety specifically in CHD adults, based on 22 studies with a total of 3,723 CHD patients; (11) the second was a systematic review focusing on depression and anxiety in adults with history of different childhood diseases, including CHD, compared to healthy controls (12). Specific to our interest, the latter pooled data from two studies of CHD patients (*n* = 999 patients vs. 229 healthy controls). In the first review, differences in the depression/anxiety symptom score were presented using Hedge’s g, which is the difference in the mean scores (comparing CHD to non-CHD adults) divided by the pooled standard deviation (SD) from the two groups. Thus, if the SDs in CHD and non-CHD participants were similar within each study included in the meta-analysis the result would be equivalent to a standardized differences in means (SMD). The overall difference in mean symptom scores suggested those with CHD had more symptoms on average [SMD = −0.11 (95%CI –0.28 to 0.06)], though this estimate is imprecise with wide confidence intervals (Figure 3). There was evidence of between study heterogeneity for the overall pooled result (I^2^ = 94%), which appeared to be influenced by disease complexity. Lesion complexity was divided into simple, moderate and great, using classifications outlined by the American College of Cardiology/American Heart Association 2008 guidelines (13). In subgroup analyses based on lesion complexity, when compared to the non-CHD peers, patients with simple or moderate lesions showed lower level of depression and anxiety symptoms, whereas patients with great complexity lesions were more likely to have higher symptom scores. The ROBIS tool indicated that there was risk of bias related to the lack of clear outcome definition, particularly on how a continuous measure across different scores was generated (11). In the second review, which pooled two studies to assess associations with binary mental health outcomes, patients with CHD had a higher odds of depression [OR = 1.63 (0.39, 6.77) and anxiety (OR = 2.58 (1.45, 4.59)] and, to a weaker extent, unspecified emotional problem (Figure 4). However, the small sample size meant results were imprecise, with wide confidence intervals and, with just two studies, it was not possible to explore between study heterogeneity. According to the ROBIS tool, there was low risk of bias.

### Cardiac Transplant

**FIGURE 5:**
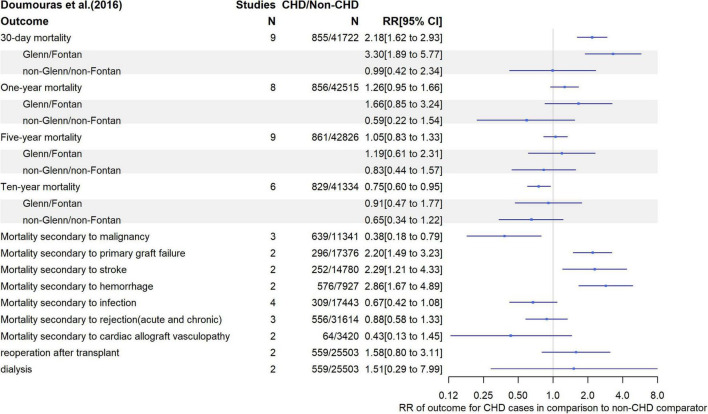
Relative risk of mortality, repeat operation and need for renal dialysis following cardiac transplantation in CHD patients compare with patients receiving transplant for other diseases.

A systematic review and meta-analysis of nine studies (*N* = 861 CHD patients) compared mortality and morbidity between adult CHD patent recipients of a cardiac transplantation and patients with other (non-CHD) diseases who had received a cardiac transplant. It found that CHD patients had a higher short term risk of mortality (RR for 30-day mortality = 2.18; 95% CI, 1.62 to 2.93) which decreased over time such that there was an apparent lower 10-year risk of death (RR = 0.75; 95% CI, 0.60 to 0.95) compared with non-CHD patients after cardiac transplantation; it was hypothesized that the latter was likely due to survivor bias (i.e., overall mortality potentially higher in CHD patients but as larger numbers die soon after the operation there are fewer living to 10 years). In subgroup analyses CHD patients who had undergone Fontan or Glenn operation were at higher risk of short-term mortality than those with other CHDs, and the short-term risk of death was higher in subgroups where death was related to primary graft failure, stroke and hemorrhage (Figure 5). According to the ROBIS tool, we found low risk of bias (Supplementary Table 2).

### Quality of Life

**FIGURE 6:**
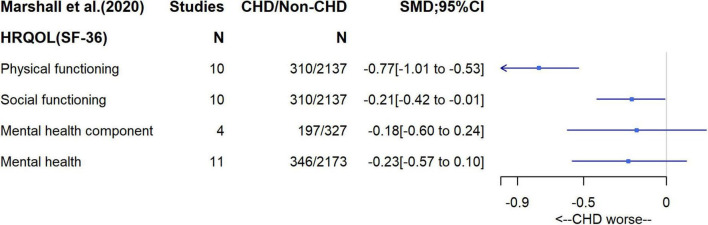
Standardize mean difference for health-related quality of life domains (SF-36).

The review of differences in QoL between CHD patients with a Fontan circulation and healthy controls included between four and eleven studies depending on which outcome was investigated (14). While the original review included studies on both children and adults, we only reported studies on adults (*N* = 346). SF-36 was used to assess QoL in all of those studies. SF-36 measures a set of generic, coherent, and easily administered QoL domains (summarized in Supplementary Figure 1) (15). Compared to healthy controls, the review found that CHD Fontan patients had reduced scores in all domains with standardized differences in means (SMD) of –0.77 (95% CI, –1.01 to –0.53), –0.21 (–0.42 to –0.01), –0.23 (–0.57 to 0.12), and –0.18 (–0.60 to 0.24) for the physical, social, mental health (i.e., anxiety and depression) domains and mental health score (a combination of vitality, social functioning, emotional role and mental health), respectively (Figure 6). Between study heterogeneity was high (I^2^ = 67%, 45%, 82%, 80% for physical, social, mental health domains and mental health component, respectively). Meta-regression suggested a tendency toward the poorer physical function in CHD patients being stronger in male patients (difference in SMD = −0.041 per% of males participating in the study; 95% CI, −0.075 to −0.007) and patients who underwent Fontan operation at later stage having lower mental health (difference in SMD = −0.25 per 1 year increase, 95% CI, −0.31 to −0.136).

The majority of the ROBIS tool criteria were met with the exception of including only English articles; however, this was acknowledged by authors (Supplementary Table 2).

## Discussion

The present umbrella review aimed to compare health and well-being in adults with CHD compared to adults without CHD. We identified only five systematic reviews that compared CHD to non-CHD, with these showing increased risk of cardiovascular diseases, depression/anxiety symptoms, 30-day and 1-year mortality after cardiac transplantation, and poorer quality of life, across physical, social and mental health domains compared to adults without CHD. Notably, we did not identify systematic reviews of several key outcomes that have been identified by patients, their families and health professionals as important, such as comorbidities (e.g., lung disease, liver disease, cancer), behaviors (e.g., physical activity, sex, contact sport), and social outcomes (e.g., education, careers). Perhaps unsurprisingly, patients with CHD were reported to have higher incidence of CVD in their adult life (10). In particular, they experienced a higher rate of heart failure. This conclusion is supported by a recent United Kingdom Biobank study that compared 2,006 adults with CHD to 497,983 without CHD and found a higher risk of major adverse cardiovascular events in the CHD patients, even after adjustment for a wide range of common cardiovascular risk factors (16). The mechanisms underlying these associations are unclear, as is the extent to which risk emerges in early life. Whilst findings from the United Kingdom Biobank study are broadly consistent with the systematic review included in this umbrella review, the findings may be biased by selection, given that only 5.5% of those who were invited to United Kingdom Biobank participated and they are known to be a healthy sub-sample of the population from which they were drawn (17).

Research on CHD patients has traditionally focused on children while little attention has been paid in the past to adult survivors. With improvements in peri-operative management, the number of children with CHD who reach adult life has increased dramatically and this has prompted interest and research on aspects of adult life in this challenging group of patients. It remains unclear whether adult life of CHD-corrected patients is comparable to their non-CHD peers and, if not, which areas should be addressed by clinicians and research.

The present umbrella review has shown that there is still little evidence on this subject, and the existing evidence is mainly focused on traditional outcomes (e.g., CVD outcomes, QoL) and there is still a lack of insights on many aspects of life among adults with CHD such as reproductive capacity, educational attainment and career prospects. The James Lind Alliance, a national non-profit making initiative, has launched an initiative to identify research priorities in CHD by bringing together patients, carers and clinicians and results will be soon available (5). Previous initiatives, such as the Working Group on adult CHD research supported by the National Heart, Lung and Blood Institute (4) has identified reproductive outcomes (e.g., the safety of pregnancy in women with CHD and potential impact on their offspring’s health and survival) (18). The present review, by highlighting the topics where there is lack of evidence, can be used as guidance for similar initiatives by highlighting what is already known and what remains unknown.

### Limitations

The nature of an umbrella review is to review and summarize evidence from published systematic reviews. However, this means that we cannot distinguish between there being very little or no research for outcomes, such as reproductive health for which we found no systematic reviews, and there being some research that has not yet been systematically reviewed. Thus, in relation to such outcomes our recommendation would be for researchers to develop systematic review protocols and undertake the systematic searches (and full reviews where relevant studies are identified) so that primary research can be directed to areas of relevance to patients and other stakeholders for which there is currently little research. One of the main problems in studying long-term outcomes of patients with CHD is the tracking of these patients. Many patients are lost in the transition between childhood and adulthood, and this may have influenced individual studies’ findings.

A key limitation of any research in this area is survivor bias. Although survival in CHD patients has progressively increased, patients with complex lesions still present lower survival rates compared to the general population (19). The proportion of deaths related to CHD among children under 1 year of age has fallen from 60–70% in the 1960s to 20–30% in 2000 (20), and this can affect comparisons with their adult non-CHD peers by masking underlying differences. The rarity of CHDs in the general population (∼1%) represents a possible obstacle in reaching meaningful conclusions from individual cohorts. To overcome these limitations, national and international collaborative health record-linkage studies, large cohorts with relevant data, such as the United Kingdom Biobank, and birth cohort collaborations such as the LifeCycle project (21) have the potential to address questions about future health and well-being in CHD patients and we would urge funders and researchers to explore and exploit these opportunities.

## Conclusion

Even though most children with CHD now reach adulthood, (20) our umbrella review identifies major gaps in the evidence around the health and other problems that patients with CHD and their families have highlighted as research priorities. Further insights into relevant aspects of adult life among CHD patients could be gained by analyzing available large prospective collaborative record-linkage and birth cohort studies (25, 26).

## Data Availability Statement

The original contributions presented in this study are included in the article/Supplementary Material, further inquiries can be directed to the corresponding author.

## Author Contributions

LC: planning, conduct, and reporting of the work described in the article, and being responsible for the overall content as guarantor, and attests that all listed authors meet authorship criteria. KT: conduct and reporting of the work described in the article. MC: reporting of the work described in the article. RC: planning, conduct, and reporting of the work described in the article. DL: planning, conduct, and reporting of the work described in the article, and being responsible for the overall content as guarantor. All authors contributed to the article and approved the submitted version.

## Author Disclaimer

The views expressed in this publication are those of the authors and not necessarily those of the United Kingdom National Health Service, the National Institute for Health Research or the United Kingdom Department of Health and Social Care, or any other funders mentioned here.

## Conflict of Interest

DL has received funding from Wellcome, the European Research Council (ERC Advanced grant and a Horizon 2020 grant), US National Institute of Health, Diabetes UK, Roche Diagnostics, and Medtronic Ltd., for research unrelated to that presented here. MC has received funding from Medtronic Ltd., for research unrelated to that presented. The remaining authors declare that the research was conducted in the absence of any commercial or financial relationships that could be construed as a potential conflict of interest.

## Publisher’s Note

All claims expressed in this article are solely those of the authors and do not necessarily represent those of their affiliated organizations, or those of the publisher, the editors and the reviewers. Any product that may be evaluated in this article, or claim that may be made by its manufacturer, is not guaranteed or endorsed by the publisher.
