# Supplementary material for: Health and Well-Being in Surviving Congenital Heart Disease Patients: An Umbrella Review With Synthesis of Best Evidence
Source: Front Cardiovasc Med. 2022 Jun 10;9:870474. doi: 10.3389/fcvm.2022.870474 (PMC9226339; doi:10.3389/fcvm.2022.870474)

**Supplementary Appendix 1. Search strategy adapted for searching in MEDLINE, EMBASE, PSYCINFO, Scopus and Cochrane library**

**MEDLINE**

((Systematic Review [All]) or (meta-analysis[All])) and ((tetralogy of Fallot [TIAB]) or ( pulmonary stenosis [TIAB]) OR (pulmonary valvar stenosis[TIAB]) or (congenital heart disease[TIAB]) or (congenital heart [TIAB])or (congenital cardiac disease [TIAB]) or (congenital heart defect [TIAB]) or (congenital heart malformation [TIAB]) or (ACHD [TIAB]) OR (GUCH [TIAB)] or (fontan circulation [TIAB]) or (cavo-pulmonary connection [TIAB]) or (univentricular heart [TIAB]) or (hypoplastic left heart syndrome [TIAB]) or (single ventricle [TIAB]) or (Norwood Procedure [TIAB]) or (double inlet left ventricle [TIAB]) or ( double outlet right ventricle [TIAB]) or (Truncus arteriosus [TIAB]) or (ebstein [TIAB]) or (tricuspid atresia [TIAB]) or (ventricular septal defect [TIAB]) or (atrial septal defect [TIAB]) or (transposition of great arteries [TIAB]) or (transposition of great vessels[TIAB]) or (arterial switch [TIAB]) or (Senning [TIAB]) or (Mustard [TIAB]) or (aortic coarctation [TIAB])or (Interrupted aortic arch [TIAB])or ( atrioventricular septal defect[TIAB]) or (total anomalous pulmonary venous connection [TIAB]) or (partial anomalous pulmonary venous connection [TIAB]) or (TAPVC [TIAB]) or (Cor triatriatum [TIAB]) or (Ross [TIAB]) or (Anomalous coronary artery [TIAB]) or (patent ductus arteriosus [TIAB] ))

**EMBASE search for the following query in the title and abstract field**

Search 1: Systematic Review

Search 2: meta-analysis

Search3: tetralogy Fallot or pulmonary stenosis or pulmonary valvar stenosis or congenital heart disease or congenital heart or congenital cardiac disease or congenital heart defect or congenital heart malformation or ACHD or GUCH or fontan circulation or cavo-pulmonary connection or univentricular heart or hypoplastic left heart syndrome or single ventricle or Norwood Procedure or double inlet left ventricle double outlet right ventricle or Truncus arteriosus or ebstein or tricuspid atresia or ventricular septal defect or atrial septal defect or transposition of great arteries or transposition of great vessels or arterial switch or Senning or Mustard or aortic coarctation or Interrupted aortic arch atrioventricular septal defect or total anomalous pulmonary venous connection or partial anomalous pulmonary venous connection or TAPVC or Cor triatriatum or Ross or Anomalous coronary artery or patent ductus arteriosus

Search 4: 1 or 2

Search 5: 3 and 4

**SCOPUS search for the following query in the abstract and title field**

( TITLE ( systematic  AND review )  OR  TITLE ( meta-analysis )  AND  TITLE ( tetralogy  AND fallot )  OR  TITLE ( pulmonary  AND stenosis )  OR  TITLE ( pulmonary  AND valvar  AND stenosis )  OR  TITLE ( congenital  AND heart  AND disease )  OR  TITLE ( congenital  AND heart )  OR  TITLE ( congenital  AND cardiac  AND disease )  OR  TITLE ( congenital  AND heart  AND defect )  OR  TITLE ( congenital  AND heart  AND malformation )  OR  TITLE ( fontan  AND circulation )  OR  TITLE ( cavopulmonary  AND connection )  OR  TITLE ( univentricular  AND heart )  OR  TITLE ( hypoplastic  AND left  AND heart  AND syndrome )  OR  TITLE ( single  AND ventricle )  OR  TITLE ( norwood  AND procedure )  OR  TITLE ( double  AND inlet  AND left  AND ventricle )  OR  TITLE ( double  AND outlet  AND right  AND ventricle )  OR  TITLE ( truncus  AND arteriosus )  OR  TITLE ( ebstein )  OR  TITLE ( tricuspid  AND atresia )  OR  TITLE ( ventricular  AND septal  AND defect )  OR  TITLE ( atrial  AND septal  AND defect )  OR  TITLE ( transposition  AND of  AND great  AND arteries )  OR  TITLE ( transposition  AND of  AND great  AND vessels )  OR  TITLE ( arterial  AND switch )  OR  TITLE ( aortic  AND coarctation )  OR  TITLE ( interrupted  AND aortic  AND arch )  OR  TITLE ( atrioventricular  AND septal  AND defect )  OR  TITLE ( total  AND anomalous  AND pulmonary  AND venous  AND connection )  OR  TITLE ( partial  AND anomalous  AND pulmonary  AND venous  AND connection )  OR  TITLE ( cor  AND triatriatum )  OR  TITLE ( anomalous  AND coronary  AND artery )  OR  TITLE ( patent  AND ductus  AND arteriosus )  OR  TITLE ( anomalous  AND coronary  AND artery )  OR  TITLE ( mustard )  OR  TITLE ( ross ) )

**Cochrane library search for the following query in the abstract and title field**

(tetralogy of Fallot or pulmonary stenosis or pulmonary valvar stenosis or congenital heart disease or congenital heart or congenital cardiac disease or congenital heart defect or congenital heart malformation or ACHD or CHD or GUCH or fontan circulation or cavo-pulmonary connection or univentricular heart or hypoplastic left heart syndrome or single ventricle or Norwood Procedure or double inlet left ventricle double outlet right ventricle or Truncus arteriosus or ebstein or tricuspid atresia or ventricular septal defect or atrial septal defect or transposition of great arteries or transposition of great vessels or arterial switch or Senning or Mustard or aortic coarctation or Interrupted aortic arch atrioventricular septal defect or total anomalous pulmonary venous connection or partial anomalous pulmonary venous connection or TAPVC or Cor triatriatum or Ross or Anomalous coronary artery or patent ductus arteriosus)

**PSYCinfo search for the following query in the abstract and title field**

Search 1: Systematic Review

Search 2: meta-analysis

Search 3: tetralogy Fallot or pulmonary stenosis or pulmonary valvar stenosis or congenital heart disease or congenital heart or congenital cardiac disease or congenital heart defect or congenital heart malformation or ACHD or GUCH or fontan circulation or cavo-pulmonary connection or univentricular heart or hypoplastic left heart syndrome or single ventcle or Norwood Procedure or double inlet left ventricle double outlet right ventricle or Truncus arteriosus or ebstein or tricuspid atresia or ventricular septal defect or atrial septal defect or transposition of great arteries or transposition of great vessels or arterial switch or Senning or Mustard or aortic coarctation or Interrupted aortic arch atrioventricular septal defect or total anomalous pulmonary venous connection or partial anomalous pulmonary venous connection or TAPVC or Cor triatriatum or Ross or Anomalous coronary artery or patent ductus arteriosus

Search 4: 1 or 2

Search 5: 4 and 3

**Supplementary appendix 2.**

Embase <1974 to 2021 July 13>

Ovid Emcare <1995 to 2021 Week 27>

Ovid MEDLINE(R) ALL <1946 to July 13, 2021>

1 "Tetralogy of Fallot"/ 22415

2 "Tetralogy of Fallot".mp. [mp=ti, ab, hw, tn, ot, dm, mf, dv, kw, fx, dq, nm, kf, ox, px, rx, ui, sy] 27279

3 exp Pulmonary Valve Stenosis/ 17393

4 (Pulmonary adj3 Stenosis).mp. [mp=ti, ab, hw, tn, ot, dm, mf, dv, kw, fx, dq, nm, kf, ox, px, rx, ui, sy] 30216

5 exp Heart Defects, Congenital/ 327071

6 (congenital adj3 heart).mp. [mp=ti, ab, hw, tn, ot, dm, mf, dv, kw, fx, dq, nm, kf, ox, px, rx, ui, sy] 194896

7 (congenital adj3 cardiac).mp. [mp=ti, ab, hw, tn, ot, dm, mf, dv, kw, fx, dq, nm, kf, ox, px, rx, ui, sy] 16150

8 ACHD.mp. [mp=ti, ab, hw, tn, ot, dm, mf, dv, kw, fx, dq, nm, kf, ox, px, rx, ui, sy] 2389

9 GUCH.mp. [mp=ti, ab, hw, tn, ot, dm, mf, dv, kw, fx, dq, nm, kf, ox, px, rx, ui, sy] 410

10 *Fontan Procedure/ 7937

11 fontan circulation.mp. [mp=ti, ab, hw, tn, ot, dm, mf, dv, kw, fx, dq, nm, kf, ox, px, rx, ui, sy] 3702

12 cavo-pulmonary connection.mp. [mp=ti, ab, hw, tn, ot, dm, mf, dv, kw, fx, dq, nm, kf, ox, px, rx, ui, sy] 314

13 exp Univentricular Heart/ 7857

14 univentricular heart.mp. [mp=ti, ab, hw, tn, ot, dm, mf, dv, kw, fx, dq, nm, kf, ox, px, rx, ui, sy] 2463

15 (univentricular adj3 heart).mp. [mp=ti, ab, hw, tn, ot, dm, mf, dv, kw, fx, dq, nm, kf, ox, px, rx, ui, sy] 2623

16 exp Hypoplastic Left Heart Syndrome/ 10873

17 "hypoplastic left heart syndrome".mp. [mp=ti, ab, hw, tn, ot, dm, mf, dv, kw, fx, dq, nm, kf, ox, px, rx, ui, sy] 12925

18 single ventricle.mp. [mp=ti, ab, hw, tn, ot, dm, mf, dv, kw, fx, dq, nm, kf, ox, px, rx, ui, sy] 14015

19 *Heart Septal Defects, Atrial/ or *Heart Septal Defects, Ventricular/ or *Heart Septal Defects/ 23740

20 "double inlet left ventricle".mp. [mp=ti, ab, hw, tn, ot, dm, mf, dv, kw, fx, dq, nm, kf, ox, px, rx, ui, sy] 754

21 "double outlet right ventricle".mp. [mp=ti, ab, hw, tn, ot, dm, mf, dv, kw, fx, dq, nm, kf, ox, px, rx, ui, sy] 4666

22 exp Truncus Arteriosus/ 2774

23 "Truncus arteriosus".mp. [mp=ti, ab, hw, tn, ot, dm, mf, dv, kw, fx, dq, nm, kf, ox, px, rx, ui, sy] 4337

24 Ebstein Anomaly/ 6559

25 ebstein.mp. [mp=ti, ab, hw, tn, ot, dm, mf, dv, kw, fx, dq, nm, kf, ox, px, rx, ui, sy] 7832

26 *Tricuspid Atresia/ 1338

27 "tricuspid atresia".mp. [mp=ti, ab, hw, tn, ot, dm, mf, dv, kw, fx, dq, nm, kf, ox, px, rx, ui, sy] 3472

28 "ventricular septal defect*".mp. [mp=ti, ab, hw, tn, ot, dm, mf, dv, kw, fx, dq, nm, kf, ox, px, rx, ui, sy] 33637

29 "atrial septal defect*".mp. [mp=ti, ab, hw, tn, ot, dm, mf, dv, kw, fx, dq, nm, kf, ox, px, rx, ui, sy] 28330

30 "Transposition of Great Vessels"/ 14582

31 "transposition of great vessels".mp. [mp=ti, ab, hw, tn, ot, dm, mf, dv, kw, fx, dq, nm, kf, ox, px, rx, ui, sy] 7928

32 "transposition of great arteries".mp. [mp=ti, ab, hw, tn, ot, dm, mf, dv, kw, fx, dq, nm, kf, ox, px, rx, ui, sy] 2437

33 "arterial switch".mp. [mp=ti, ab, hw, tn, ot, dm, mf, dv, kw, fx, dq, nm, kf, ox, px, rx, ui, sy] 5441

34 Senning.mp. [mp=ti, ab, hw, tn, ot, dm, mf, dv, kw, fx, dq, nm, kf, ox, px, rx, ui, sy] 1769

35 *Aortic Coarctation/ 8909

36 Aortic Coarctation.mp. [mp=ti, ab, hw, tn, ot, dm, mf, dv, kw, fx, dq, nm, kf, ox, px, rx, ui, sy] 18472

37 "Interrupted aortic arch".mp. [mp=ti, ab, hw, tn, ot, dm, mf, dv, kw, fx, dq, nm, kf, ox, px, rx, ui, sy] 2712

38 atrioventricular septal defect.mp. [mp=ti, ab, hw, tn, ot, dm, mf, dv, kw, fx, dq, nm, kf, ox, px, rx, ui, sy] 5686

39 "total anomalous pulmonary venous connection".mp. [mp=ti, ab, hw, tn, ot, dm, mf, dv, kw, fx, dq, nm, kf, ox, px, rx, ui, sy] 1991

40 "partial anomalous pulmonary venous connection".mp. [mp=ti, ab, hw, tn, ot, dm, mf, dv, kw, fx, dq, nm, kf, ox, px, rx, ui, sy] 1128

41 TAPVC.mp. [mp=ti, ab, hw, tn, ot, dm, mf, dv, kw, fx, dq, nm, kf, ox, px, rx, ui, sy] 980

42 *Cor Triatriatum/ 1767

43 Cor triatriatum.mp. [mp=ti, ab, hw, tn, ot, dm, mf, dv, kw, fx, dq, nm, kf, ox, px, rx, ui, sy] 2681

44 *Coronary Vessel Anomalies/ 9381

45 Anomalous coronary artery.mp. [mp=ti, ab, hw, tn, ot, dm, mf, dv, kw, fx, dq, nm, kf, ox, px, rx, ui, sy] 1424

46 patent ductus arteriosus.mp. [mp=ti, ab, hw, tn, ot, dm, mf, dv, kw, fx, dq, nm, kf, ox, px, rx, ui, sy] 32935

47 1 or 2 or 3 or 4 or 5 or 6 or 7 or 8 or 9 or 10 or 11 or 12 or 13 or 14 or 15 or 16 or 17 or 18 or 19 or 20 or 21 or 22 or 23 or 24 or 25 or 26 or 27 or 28 or 29 or 30 or 31 or 32 or 33 or 34 or 35 or 36 or 37 or 38 or 39 or 40 or 41 or 42 or 43 or 44 or 45 or 46 439945

48 ((emotion* or psychosocial or psychological) adj2 (problem* or function* or symptom* or issue* or outcome* or impact or distress)).mp. [mp=ti, ab, hw, tn, ot, dm, mf, dv, kw, fx, dq, nm, kf, ox, px, rx, ui, sy] 297553

49 "Quality of Life"/ 904750

50 ("quality of life" or "health related quality of life" or QoL or HRQoL).mp. [mp=ti, ab, hw, tn, ot, dm, mf, dv, kw, fx, dq, nm, kf, ox, px, rx, ui, sy] 1253659

51 ((psychosocial or psychological) adj2 (outcome* or impact or function* symptom* or distress)).mp. [mp=ti, ab, hw, tn, ot, dm, mf, dv, kw, fx, dq, nm, kf, ox, px, rx, ui, sy] 115945

52 Psychosocial Functioning/ 114465

53 "Mental Health".mp. [mp=ti, ab, hw, tn, ot, dm, mf, dv, kw, fx, dq, nm, kf, ox, px, rx, ui, sy] 694767

54 *Mental Health/ 106146

55 depression.mp. [mp=ti, ab, hw, tn, ot, dm, mf, dv, kw, fx, dq, nm, kf, ox, px, rx, ui, sy] 1376007

56 Depression/ 629680

57 (anxi* or worr* or "low mood" or "feeling down").mp. [mp=ti, ab, hw, tn, ot, dm, mf, dv, kw, fx, dq, nm, kf, ox, px, rx, ui, sy] 911126

58 Anxiety/ 403021

59 (life* adj2 (change* or challenge*)).mp. [mp=ti, ab, hw, tn, ot, dm, mf, dv, kw, fx, dq, nm, kf, ox, px, rx, ui, sy] 82600

60 ((chronic or serious) adj2 (ill* or mobidit*)).mp. [mp=ti, ab, hw, tn, ot, dm, mf, dv, kw, fx, dq, nm, kf, ox, px, rx, ui, sy] 93063

61 48 or 49 or 50 or 51 or 52 or 53 or 54 or 55 or 56 or 57 or 58 or 59 or 60 3734458

62 47 and 61 14118

63 limit 62 to yr="2015 - 2021" 6362

**Supplementary Appendix 3. Data extraction tools**

| **Author, year** | **Study Population** | **Outcome Type** | **Number of study (k)** | **Definition of Outcome** | **Sample size (CHD/non-CHD)** | **Main result**  **[95%CI], Fixed or random effect** | **Subgroup analysis /Confounding/source of bias** | **Heterogeneity** |
| --- | --- | --- | --- | --- | --- | --- | --- | --- |
| Marshall KH 2020(25) | Fontan,  mean patient age at SF-36  assessment  ranged from 20.7 to 27 years | QoL | Physical functioning  k=10  Social functioning  k=10  Mental health component  k=4  Mental health  k=11 | SF-36 | Physical functioning  310 VS 2137  Social  310 VS 2137  Mental health component  197 VS 327  Mental health  346 VS 2173 | Standardized mean difference (SMD), Random effect  Physical functioning  SMD −0.77; 95% CI, −1.01 to −0.53  Social functioning  SMD −0.21; 95% CI, −0.42 to −0.01  Mental health component  SMD −0.18; 95% CI,−0.60 to 0.24;  Mental health  SMD−0.23; 95% CI, −0.57 to 0.12 | older age at Fontan operation was associated with lower self-reported mental health scores  (ß=0.225; 95% CI, -0.314 to -0.136; P<0.0001).  Studies with a higher proportion of female patients yielded a smaller difference in self-reported physical  (ß=-functioning compared with referents 0.041; 95% CI, -0.075 to -0.007; P=0.018).  No mention of confounder adjustment in the included studies. | Physical functioning  I^2^=66.64  Social  I^2^=45.39  Mental health component  I^2^=80.06  Mental health  I^2^=82.41 |
| Wang T, 2019(24) | CHD,  6 out of 9 studies were adults only, 2 studies were a mix of children and adults and 1 study included just children  **(FARAONI)** | Risk of Cardiovascular disease | 9 | CVD  Stroke  Heart failure  Coronary heart disease  Cardiac arrest | 81137 CHD  Vs  603063 NO CHD | Fixed effect  CVD RR 3.12  [3.01-3.24]  Stroke RR 2.46  [2.30– 2.63]  Heart failure RR 5.89  [5.58–6.21]  Coronary HD RR 1.5  [1.40– 1.61] | Overall, individuals with CHD were still at a higher risk of CVD among all subgroup data. Specifically, when data were restricted to studies conducted in European countries (4.25; 95% CI, 4.06–4.45), studies with a prospective cohort design (4.79; 95% CI, 4.56–5.04), studies with samples from the general population (4.12; 95% CI, 3.94–4.30), studies included adult and child survivors (10.15; 95% CI, 8.07–12.77), and studies only adjusted for age and sex (4.81; 95% CI, 4.59–5.04), the risk of developing CVD associated with CHD increased further.  All included studies adjusted for at minimum age and sex. One study additionally adjusted for education and history of other chronic illness, one study additionally adjusted for smoking status and one study additionally adjusted for race. Authors conclude that they cannot determine whether the associations are confounded by a risk factor profile of CVD among CHD survivors or whether CHD is an independent risk factor. | CVD  I2 =99%  Stroke  I2= 97%  Heart failure I2=93%  Coronary HD I2=91% |
| Jackson JL, 2015(19) | CHD  2 out of the 22 studies included adolescents only | Emotional Functioning | 22 | Symptoms of depression and anxiety | 3723 CHD  Vs  NO CHD compared to norms data,  Studies ranged from 27 cases to 767 cases of CHD. | Random effect  CHD did not report greater emotional distress than controls/norms  (Hedge’s g= -0.11 [ −0.28 to 0.06]) | Survivors with complex lesions reported similar levels of emotional functioning as control or normative data (Hedges’ g = −0.07, 95% CI: −0.34 to 0.120, p = 0.596).  Moderate lesions reported significantly better emotional functioning than controls or measure norms (Hedge’s g = 0.28, 95% CI: 0.09 to 0.47, p = 0.004).  A trend was identified for survivors of CHD with simple lesions to report better emotional functioning than controls/norms (Hedge’s g = 0.21, 95% CI: −0.01 to 0.43, p = 0.064).  No differences in effect sizes were found as a function of the Gini Index scores reflecting levels of income distribution equality using random effects models in either meta-regression (β = 0.41, SE = 0.43, p = 0.33) or categorical analysis (Q statistic = 0.20, p = 0.65).  No mention of confounder adjustment in the included studies. | I2 = 94%  Q stat = p <0.001 |
| Secinti E, 2017 | CHD,  This paper focused on childhood chronic illness and included CHD as a subtype. | Emotional problem | unipolar depressive episode, major depres-  sion, generalised anxiety disorder, panic disorder, phobias,  social anxiety disorder, posttraumatic stress disorder (PTSD),  obsessive-compulsive disorder (OCD), or health anxiety  unipolar depressive episode, major depres-  sion, generalised anxiety disorder, panic disorder, phobias,  social anxiety disorder, posttraumatic stress disorder (PTSD),  obsessive-compulsive disorder (OCD), or health anxiety  unipolar depressive episode, major depres-  sion, generalised anxiety disorder, panic disorder, phobias,  social anxiety disorder, posttraumatic stress disorder (PTSD),  obsessive-compulsive disorder (OCD), or health anxiety  K=2 | Depression  Anxiety  Unspeciﬁed emotional problems | 999 CHD vs 229 no CHD | Depression  OR=1.63 [0.39, 6.77]  Anxiety  OR=2.58 [1.45, 4.59]  Unspeciﬁed emotional problems  OR= 1.18 [0.77, 1.82] | Possible moderators explored using meta regression analyses but not done for chronic illness subtypes.  No mention of confounder adjustment in the included studies. | Not reported for CHD |
| Doumouras BS 2016(26) | CHD  (age > 18) | Post-transplant outcomes | 30-day mortality  K=9  One-year mortality  K=8  Five-year mortality  K=9  Ten-year mortality  K=6  Mortality secondary to malignancy  K=3  Mortality secondary to primary graft failure  K=2  Mortality secondary to stroke  K=2  Mortality secondary to haemorrhage  K=2 | 30-day mortality  One-year mortality  Five-year mortality  Ten-year mortality  Cause of mortality  Reoperation and risk of dialysis | 30-day mortality  855 CHD VS 41,722 no CHD  One-year mortality  856 CHD VS 42515 no CHD  Five-year mortality  861 CHD VS 42826 no CHD  Ten-year mortality  829 CHD VS 41334 no CHD  Mortality secondary to malignancy  639 CHD and 11,341 no CHD  Mortality secondary to primary graft failure  296 CHD and 17,376 no CHD  Mortality secondary to stroke  252 CHD and 14,780 no CHD  Mortality secondary to haemorrhage  576 CHD and 7,927 no CHD | Random effect  30-day mortality  RR=2.18; 95%CI,1.62–2.93  One-year mortality  RR=1.26; 95%CI,0.95–1.66  Five-year mortality  RR=1.05;95%CI,0.83–1.33  Ten-year mortality  RR=0.75;95%CI,0.60–0.95  Mortality secondary to malignancy  RR, 0.38;95%CI,0.18–0.79  Mortality secondary to primary graft failure  RR,2.20;95%CI,1.49–3.23  Mortality secondary to stroke  RR, 2.29;95%CI,1.21–4.33  Mortality secondary to haemorrhage  RR,2.86,95%CI,1.67–4.89  Death due to infection, rejection, and CAV were all decreased in CHD; however, none of these outcomes achieved statistical significance  Although more CHD patients required reoperation (21.1%vs10.5%) and dialysis (21.5%vs8.4%), neither outcome was statistically significant | Increased early mortality risk in those with a prior Fontan or Glenn operation.  (43.8% CHD vs 14.4% NO CHD RR, 3.30;95%CI,1.89–5.77; I2 = 0%), whereas the risk at the remaining times was non-significant.  The CHD single-ventricle patients without previous Fontan/Glenn procedures had similar mortality out comes as non-CHD patients (RR,0.99, 95%CI ,0.42–2.34; p=0.02 for sub-group interaction test).  Studies did not consider potential confounders | 30-day mortality  I^2^ =41%  One-year mortality  I^2^ =36%  Five-year mortality  I^2^ = 45%  Ten-year mortality  I^2^ = 42%  Mortality secondary to malignancy  I^2^ =0  Mortality secondary to primary graft failure  I^2^ =21%  Mortality secondary to stroke  I^2^ = 0%  Mortality secondary to haemorrhage  I^2^ = 0 |

**Supplementary Table 1. Reasons for exclusion for each study.**

| **Reference** | **URL** | **Reason for exclusion** |
| --- | --- | --- |
| Abda 2019 | <https://academic.oup.com/jpepsy/article/44/4/463/5187995?searchresult=1> | Not adult |
| Akbar 2017 | <https://www.heartlungcirc.org/article/S1443-9506(17)31191-5/fulltext> | Conference abstract |
| Alnasser 2018 | <https://www.internationaljournalofcardiology.com/article/S0167-5273(17)37507-1/fulltext> | No control comparison group |
| Alsaied 2017 | <https://onlinelibrary.wiley.com/doi/abs/10.1111/chd.12468> | No control comparison group |
| Amianto 2013 | <https://pubmed.ncbi.nlm.nih.gov/24441518/> | Not English |
| Anderson 2017 | <https://www.ncbi.nlm.nih.gov/pmc/articles/PMC6481392/pdf/CD008895.pdf> | Not related to CHD |
| Andries 2017 | <https://pubmed.ncbi.nlm.nih.gov/29035962/> | Not in adults |
| Atashb 2015 | <http://www.eurekaselect.com/133614/article> | No control comparison group |
| Athanasiadis 2019 | <https://link.springer.com/content/pdf/10.1007/s00246-019-02133-z.pdf> | No control comparison group |
| Bai | Is percutaneous closure superior to surgical repair in terms of heart blocks after closure of perimembranous ventricular septal defect? evidence from a meta-analysis | Not full text |
| Baldessarini 2006 | <https://onlinelibrary.wiley.com/doi/epdf/10.1111/j.1399-5618.2006.00344.x> | Not related to CHD |
| Bedard 2009 | <https://pubmed.ncbi.nlm.nih.gov/19147605/> | Not a systematic review |
|  |  |  |
| Bellinger 2013 | <https://psycnet.apa.org/record/2013-09920-005v> | Not a systematic review |
| Ben 2019 | <https://www.annalsthoracicsurgery.org/action/showPdf?pii=S0003-4975%2818%2931442-5> | No control comparison group |
| Ben 2016 | <https://www.sciencedirect.com/science/article/pii/S0828282X16304688> | Conference abstract |
| Beerdajs 2014 | <https://academic.oup.com/ejcts/article/46/6/944/386850> | No control comparison group |
| Best 2016 | <https://www.ncbi.nlm.nih.gov/pmc/articles/PMC4937249/pdf/JAH3-5-e002846.pdf> | Not looking at health-related outcomes . |
| Brambilla 2014 | <https://www.physicamedica.com/article/S1120-1797(13)00454-7/fulltext> | Not adult |
| Bratt 2015 | <https://www.internationaljournalofcardiology.com/article/S0167-5273(15)01127-4/fulltext> | Not systematic review |
| Campbell 2016 | <https://pubmed.ncbi.nlm.nih.gov/27128768/> | Not looking at health-related outcomes |
| Capone 2017 | <https://onlinelibrary.wiley.com/doi/epdf/10.1002/ajmg.a.38512> | No control comparison group |
| Chong 2018 | <https://link.springer.com/article/10.1007/s00431-017-3081-y> | Not adult |
| Clancy 2019 | <https://link.springer.com/content/pdf/10.1007/s10880-019-09651-1.pdf> | Not adult |
| Cobham 2019 | <https://jaacap.org/article/S0890-8567(19)32110-0/pdf> | Not adults |
| Córdova-Palomera 2019 | <https://www.nature.com/articles/s41598-019-52969-x.pdf> | Not looking at health-related outcomes |
| Denniss 2017 | <https://www.heartlungcirc.org/article/S1443-9506(17)31189-7/pdf> | Conference abstract |
| Dulfer 2013 | <https://journals.sagepub.com/doi/pdf/10.1177/2047487313494030> | Not adult |
| Eagleson 2019 |  | Abstract |
| Etnel 2018 | <https://www.ahajournals.org/doi/epub/10.1161/CIRCOUTCOMES.118.004748> | No control comparison group |
| Feldmann 2019 | <https://systematicreviewsjournal.biomedcentral.com/articles/10.1186/s13643-019-1153-y> | Not a Systematic Review |
| Foulds 2017 | <https://www.nature.com/articles/jhh201755> | No control comparison group |
| Fteropulli et al. 2013 | <https://pubmed.ncbi.nlm.nih.gov/23388149/> | Did not meet definition of systematic review. |
| Garcia Ropero 2018 | <https://www.ahajournals.org/doi/epub/10.1161/CIRCOUTCOMES.117.004575> | No control comparison group |
| Gnanappa 2017 | <https://link.springer.com/content/pdf/10.1007/s00246-016-1484-6.pdf> | No control comparison group |
| Goossens 2010 |  | Conference abstract |
| Gregory 2018 | <https://journals.lww.com/jcnjournal/Abstract/2018/07000/Quality_of_Life_for_Parents_of_Children_With.11.aspx> | Not related to CHD |
| Hardee 2020 | <https://www.onlinejacc.org/content/75/11_Supplement_1/613> | Conference abstract |
| Harel-Sterling 2019 | <https://www.onlinejacc.org/content/73/9_Supplement_1/656> | Conference abstract |
| Hargrove 2005 | <https://link.springer.com/content/pdf/10.1007/s00246-005-0950-3.pdf> | Not looking at health-related outcomes |
| Izzo 1990 | <https://journals.sagepub.com/doi/10.1177/0093854890017001008> | Not related to CHD |
| Jackson 2015 | <https://link.springer.com/content/pdf/10.1007/s00246-015-1121-9.pdf> | Not related to CHD |
| Jackson | Children and adults with univentricular hearts score lower on full scale intelligence testing compared to children with biventricular congenital heart disease: A meta-analysis | No text found |
| Kahr et al 2015 | <https://pubmed.ncbi.nlm.nih.gov/26204568/> | No control group. |
| Kahr 2018 | <https://www.ahajournals.org/doi/abs/10.1161/circ.130.suppl_2.17395> | Conference abstract |
| Karsdorp 2007 | <https://academic.oup.com/jpepsy/article/32/5/527/930328> | Not adult |
| Ladak 2017 | <https://academic.oup.com/eurheartj/article/38/suppl_1/ehx493.P6321/4087337> | Abstract |
| Lane 2013 | <https://pubmed.ncbi.nlm.nih.gov/24163137/> | No results, Cochrane review found no studies pertinent to their research question |
| Latal 2009 | <https://bmcpediatr.biomedcentral.com/track/pdf/10.1186/1471-2431-9-6> | Not adult |
| Li 2019 | <https://journals.lww.com/jcrjournal/Fulltext/2019/09000/Exercise_Training_in_Adults_With_Congenital_Heart.4.aspx> | No control comparison group |
| Lip 2003 | <https://pubmed.ncbi.nlm.nih.gov/12918013/> | No results, Cochrane review found no studies pertinent to their research question |
| Ludmir 2019 | <https://scihub.wikicn.top/https://www.jpsmjournal.com/article/S0885-3924(19)30439-7/fulltext> | No control comparison group |
| Massaro 2008 | <https://www.brainanddevelopment.com/article/S0387-7604(08)00005-3/fulltext> | Not adult |
| Mongeon 2019 | <https://www.onlinecjc.ca/article/S0828-282X(19)31190-0/abstract> | No control comparison group |
| Papasavvas 2017 | <https://journals.lww.com/jcrjournal/Fulltext/2017/07000/Association_Between_Depressive_Symptoms_and.1.aspx> | Not related to CHD patients |
| Pyngottu 2019 | <https://link.springer.com/content/pdf/10.1007/s00246-018-2038-x.pdf> | No adult |
| Rigatelli 2014 | <https://www.tandfonline.com/doi/full/10.1586/14779072.5.6.1135> | Not a Systematic review |
| Shaddy 2018 | <https://www.ncbi.nlm.nih.gov/pmc/articles/PMC5829104/pdf/246_2017_Article_1787.pdf> | Not adult |
| Shen 2014 | <https://obgyn.onlinelibrary.wiley.com/doi/full/10.1002/uog.13378> | Not a Systematic review |
| Sistino 2012 | <https://www.ncbi.nlm.nih.gov/pmc/articles/PMC4557564/pdf/ject-44-216.pdf> | No control comparison group |
| Snookes 2010 | <https://pediatrics.aappublications.org/content/125/4/e818.long> | Not adult |
| Sterken 2015 | <https://openheart.bmj.com/content/openhrt/2/1/e000255.full.pdf> | Not adults |
| Stout 2018 | <https://www.ahajournals.org/doi/10.1161/CIR.0000000000000603> | Not a Systematic Review |
| Suyin 2010 | <https://pubmed.ncbi.nlm.nih.gov/27820382/> | Not related to CHD |
| Svensson 2019 | <https://journals.sagepub.com/doi/10.1177/1049732319869909> | Not looking at health-related outcomes |
| Syrgani 2016 | <http://journals.rcni.com/nursing-children-and-young-people/oc18-neurodevelopmental-outcomes-following-congenital-heart-surgery-ncyp.28.4.68.s49> | Abstract |
| Tesson 2019 | <https://pubmed.ncbi.nlm.nih.gov/30652913/> | No control comparison group |
| Venkatesh 2019 | <https://www.ahajournals.org/doi/epub/10.1161/JAHA.119.012932> | Not looking at health-related outcomes |
| Verheugt 2008 | <https://www.internationaljournalofcardiology.com/article/S0167-5273(08)00793-6/abstract> | Not looking at health-related outcomes |
| Wang 2015 | <https://onlinelibrary.wiley.com/doi/abs/10.1111/ped.12773> | Not a Systematic Review |
| Wang 2019 | <https://heart.bmj.com/content/105/21/1661.long> | No control comparison group |
| Yuan 2019 | <https://pubmed.ncbi.nlm.nih.gov/31659968/> | No control comparison group |
| Zhang 2017 | <https://link.springer.com/content/pdf/10.1007/s12519-016-0068-0.pdf> | Not adult |
| Zwijnenburg 2018 | <https://www.sciencedirect.com/science/article/abs/pii/S0002870318301042?via%3Dihub> | No control comparison group |
| Bolduc 2018 | <https://onlinelibrary.wiley.com/doi/epdf/10.1111/dmcn.13975> | Not adult |
| Huisenga 2020 | <https://onlinelibrary.wiley.com/doi/epdf/10.1111/dmcn.14512> | Not adult |
|  |  |  |

**Supplementary Table 2. Risk of bias assessments (ROBIS)**

| **Study** | **Relevance**  **(yes, no, partial)** | **Study eligibility criteria** | **Identification**  **and**  **selection of studies** | **Data collection**  **And**  **study appraisal** | **Synthesis**  **And**  **findings** | **Overall risk**  **Of**  **bias** |
| --- | --- | --- | --- | --- | --- | --- |
| Wang, 2019 | Partial | Low | High | Unclear | Low | Low |
| Marshall, 2020 | Yes | Low | High | Low | Low | Low |
| Jackson, 2015 | Yes | High | High | Unclear | Low | High |
| Secinti, 2017 | Yes | Low | Low | Unclear | Low | Low |
| Doumouras, 2016 | Yes | Low | Low | High | Low | Low |

**Supplementary Figure 1**


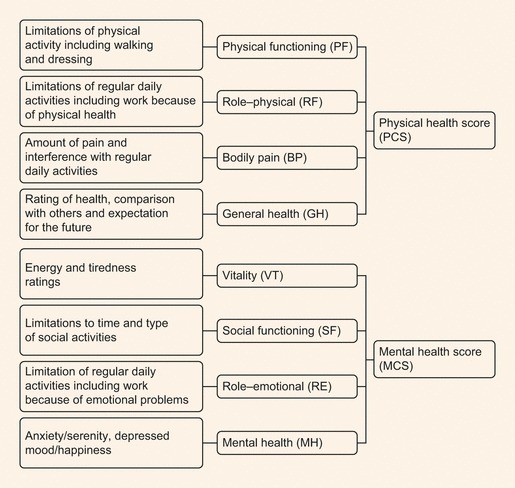

Supplement: Supplementary file 1 [file Data_Sheet_1.docx]
